# Prolonged Treatment in a Case of Anti–N‐Methyl‐D‐Aspartate Receptor Encephalitis

**DOI:** 10.1155/crog/8497402

**Published:** 2026-09-16

**Authors:** Thuy-Mai Thi Huynh, Ngoc-Trinh Thi Nguyen, Anh-Thu Thi Nguyen, Khanh-Trang Nguyen Huynh, Diem-Tuyet Thi Hoang, Thanh-Viet Pham, Ngoc-Khanh Thi Do, Quoc-Hung Le

**Affiliations:** ^1^ Hung Vuong Hospital, Ho Chi Minh City, Vietnam; ^2^ Faculty of Public Health, Pham Ngoc Thach University of Medicine, Ho Chi Minh City, Vietnam, pnt.edu.vn; ^3^ Pham Ngoc Thach University of Medicine, Ho Chi Minh City, Vietnam, pnt.edu.vn; ^4^ Cho Ray Hospital, Ho Chi Minh City, Vietnam, choray.vn; ^5^ Faculty of Medicine, Nguyen Tat Thanh University, Ho Chi Minh City, Vietnam, ntt.edu.vn

**Keywords:** anti-NMDAR IgG, anti–N-methyl-D-aspartate receptor encephalitis, plasmapheresis, teratoma

## Abstract

**Background:**

Anti–N‐methyl‐D‐aspartate receptor (anti‐NMDAR) encephalitis is a rare autoimmune encephalitis. The proposed mechanism relates to the circulating tumor‐induced antibody, so the treatment includes tumor removal and immunotherapy. This report is aimed at describing a case of anti‐NMDAR encephalitis in a female patient with an ovarian teratoma who required prolonged immunotherapy.

**Case Report:**

A 17‐year‐old female presented with a fever before hospital admission, then progressed to neurologic and psychiatric symptoms. The indirect immunofluorescence antibody (IFA) test revealed positive anti‐NMDAR immunoglobulin G (IgG) in serum and cerebrospinal fluid (CSF) samples. The patient was diagnosed with teratomas on both ovaries at different times. Two surgeries were performed to remove the tumors. The patient received systemic corticosteroids and 30 sessions of plasmapheresis. After approximately 2 months of treatment, the patient improved significantly and was discharged. At follow‐up, she showed good clinical recovery, although mild residual cognitive impairment was noted on formal assessment.

**Conclusions:**

This case highlights that, in young women with anti‐NMDAR encephalitis, early and thorough evaluation for ovarian teratoma is essential. If recovery remains incomplete after initial surgery and immunotherapy, repeat assessment for occult, residual, or contralateral lesions should be considered. Careful preoperative lesion assessment and fertility‐preserving surgical planning may help reduce missed lesions while minimizing unnecessary ovarian tissue loss.

## 1. Background

Anti–N‐methyl‐D‐aspartate receptor (anti‐NMDAR) encephalitis is one of the best characterized forms of autoimmune encephalitis and predominantly affects children and young women. Although its pathogenesis has not been fully elucidated, the disease is characterized by autoantibodies directed against neuronal N‐methyl‐D‐aspartate receptors, resulting in a broad spectrum of neuropsychiatric manifestations [1]. Ovarian teratoma has long been recognized as the most common associated neoplasm, and neural elements within the tumor are thought to trigger the production of pathogenic autoantibodies [2, 3]. Nevertheless, tumor‐associated immunity does not explain every clinical presentation, and alternative mechanisms, including intrathecal antibody synthesis and antibody passage across a disrupted blood–brain barrier, have also been proposed.

Current management therefore combines prompt immunotherapy with surgical removal of the associated tumor whenever feasible, as this strategy is associated with improved neurological recovery [1]. However, an important clinical challenge arises when patients fail to improve despite apparently appropriate treatment. The educational value of the present case lies not in the established association between anti‐NMDAR encephalitis and ovarian teratoma, but in the delayed recognition of contralateral ovarian teratomas after the initial surgery, which prolonged the disease course. The case also illustrates the need to balance complete elimination of potential antigenic tissue with preservation of future reproductive function in an adolescent patient.

## 2. Case Presentation

**Scheme 1:**
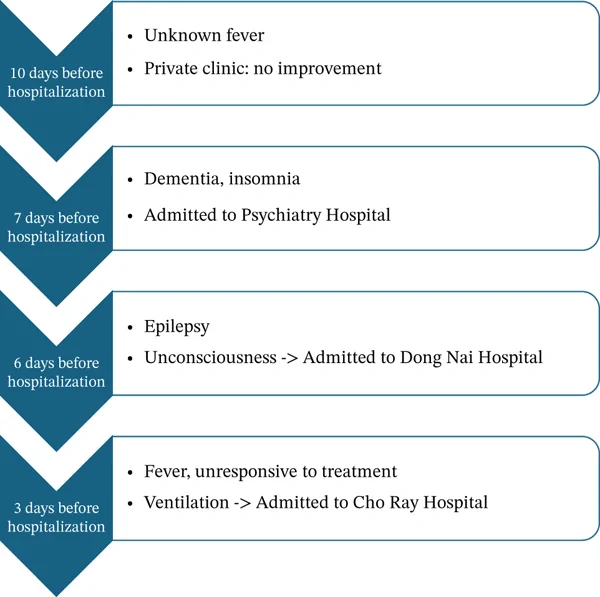
Symptom presentation in chronological order.

A 17‐year‐old girl was admitted because of altered mental status. At presentation, she was agitated, delirious, and unable to communicate appropriately. The chronological symptoms of the patient were as follows: (Scheme 1)

On examination, the Glasgow Coma Scale was at 9 pts. (the eye movement of 3 pts., the motor response of 5 pts., and the verbal response of 1 pt.). She denied any signs of infection in the respiratory, gastrointestinal, or urogenital tract. There was no history of trauma and no skin bruise or purpura. Neurological examination revealed a pupil diameter of 2.5 mm with light reflex. Neck stiffness sign was positive; Babinski signs were negative. No images of intracranial lesions were observed in the computed tomography examination. Electroencephalography showed bifocal slow wave activity. The primary diagnosis was meningoencephalitis, and the differential diagnosis was tuberculous meningitis.

**Figure 1:**
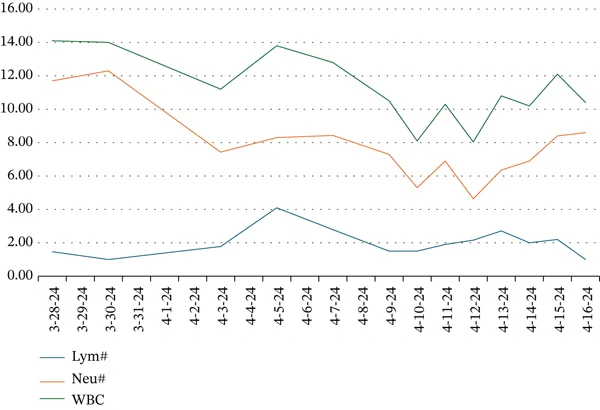
Total blood count results during the therapy.

Initial investigations focused on excluding common causes of acute encephalopathy. Neuroimaging showed no evidence of intracranial lesions or ischemic stroke, whereas microbiological analyses of CSF, blood cultures, and polymerase chain reaction assays for *Mycobacterium tuberculosis* and herpes simplex virus were all negative. These findings made infectious meningoencephalitis and cerebrovascular disease unlikely. Consequently, the diagnostic workup was expanded to include autoimmune testing. Although serum anti‐dsDNA, anti‐Sm, and antiphospholipid antibodies were negative, anti‐NMDAR IgG was detected in both serum (screening dilution 1:10) and CSF (screening dilution 1:1), confirming the diagnosis of anti‐NMDAR encephalitis (Figure 1).

Simultaneously, pelvic CT scanning detected a mass (12 × 14 mm) on the left ovary with calcified infiltration. We performed tumor removal surgery, and the pathology of the tumor was revealed as a mature teratoma. However, the patient was still febrile after the surgery despite being on antibiotics. On postoperative Day 4, the patient developed facial catatonia and seizure. After 1‐month postsurgery, the anti‐NMDAR IgG was still positive. Repeat pelvic imaging with MRI revealed two more tumors in the right ovary: 16 × 16 mm for the bigger one and 12 × 12 mm for the smaller one. This finding highlights the limitations of imaging modalities in detecting small ovarian teratomas, as lesions with subtle imaging features or small size may be overlooked on initial evaluation [4].

Because the patient was only 17 years old, planning the second operation required careful consideration of fertility preservation while ensuring complete removal of potential antigenic tissue. Histopathological examination again confirmed mature teratomas. Plasmapheresis, initiated on postoperative Day 4 after the first operation, was continued throughout the treatment course for a total of 30 sessions. Following the second surgery, the patient′s neurological condition improved steadily, and follow‐up testing demonstrated disappearance of anti‐NMDAR IgG.

**Figure 2:**
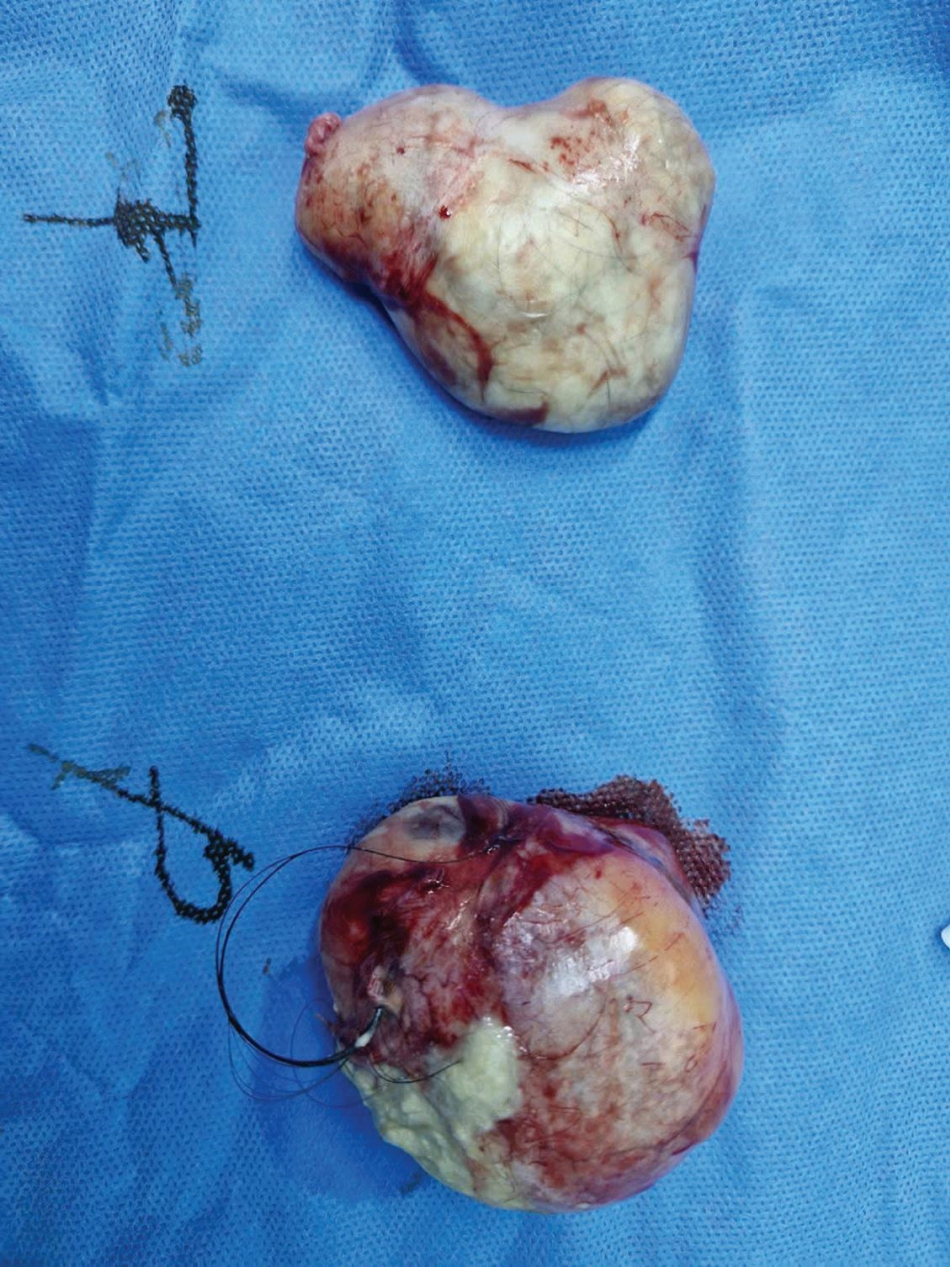
Two tumors of the right ovary from the second surgery.

After approximately 2 months of treatment, the patient was discharged with good recovery. At follow‐up in April 2025, the patient returned for re‐examination. Formal neuropsychological assessment using the WAIS‐III suggested mild residual cognitive impairment. Thus, the clinical reasoning in this case was based on persistent disease activity despite initial tumor removal and immunotherapy, which prompted repeat pelvic reassessment and led to identification of contralateral ovarian teratomas (Figure 2).

## 3. Discussion

The principal educational message of this case is not the well‐established association between anti‐NMDAR encephalitis and ovarian teratoma, but the diagnostic challenge posed by persistent disease activity after apparently adequate initial treatment. Although ovarian teratomas are recognized as the most common neoplastic association with anti‐NMDAR encephalitis [2, 3], failure to achieve the expected clinical response should prompt clinicians to reconsider whether the antigenic source has been completely identified and removed.

Anti‐NMDAR encephalitis is mediated by autoantibodies targeting the GluN1 subunit of the NMDA receptor, resulting in receptor dysfunction and a broad spectrum of neuropsychiatric manifestations [3]. Although ovarian teratomas containing differentiated neural tissue are considered the principal source of autoantibody production [2], alternative mechanisms—including receptor capping and internalization, leading to reduced surface NMDAR expression [5], as well as postinfectious immune activation [6]—have also been proposed. Consequently, comprehensive tumor screening remains an essential component of the diagnostic workup, but the absence of an obvious lesion or an incomplete response after treatment should not exclude further investigation.

Our patient fulfilled the currently accepted diagnostic criteria for anti‐NMDAR encephalitis, including rapid onset of neuropsychiatric symptoms, abnormal electroencephalography, exclusion of infectious etiologies, and detection of anti‐NMDAR IgG in both serum and cerebrospinal fluid [1]. Her demographic characteristics and clinical presentation were also consistent with previously reported cases involving young women of reproductive age [7]. However, unlike the typical clinical course in which neurological recovery follows tumor removal combined with immunotherapy [8, 9], our patient continued to deteriorate after resection of the initially identified ovarian teratoma. Persistent anti‐NMDAR IgG positivity together with ongoing neurological manifestations prompted repeat pelvic imaging, ultimately revealing two previously unrecognized mature teratomas in the contralateral ovary. This sequence strongly suggests that persistent disease activity should not automatically be attributed to delayed immunologic recovery alone.

Several factors may explain why the contralateral lesions were not detected during the initial evaluation. Small ovarian teratomas may exhibit subtle radiological features and therefore be difficult to identify on initial imaging [4]. Furthermore, when patients present with severe neuropsychiatric symptoms requiring urgent stabilization, diagnostic attention may naturally focus on neurological management rather than comprehensive gynecologic reassessment. Identification of one ovarian lesion may also create premature diagnostic closure, reducing suspicion for additional contralateral pathology. This case therefore emphasizes the importance of systematic bilateral adnexal evaluation, multidisciplinary discussion among neurologists, radiologists, gynecologists, and surgeons, and repeat pelvic imaging when the clinical response after initial treatment is not as anticipated.

Following the second operation, the patient′s neurological condition improved steadily, seizures resolved completely, and anti‐NMDAR IgG became undetectable. Although causality cannot be established from a single case, the temporal relationship between removal of the remaining teratomas and subsequent clinical recovery supports the concept that complete elimination of the antigenic source may contribute to favorable outcomes in selected patients with anti‐NMDAR encephalitis associated with ovarian teratoma. These findings remain consistent with current evidence supporting multimodal treatment combining tumor removal and immunotherapy [2, 10, 11].

An additional consideration in this case was the patient′s age. Since she was only 17 years old, repeat ovarian surgery required careful balancing between complete removal of potential antigenic tissue and preservation of future reproductive function. The decision to proceed with a second operation was based on persistent neurological disease, continued anti‐NMDAR IgG positivity, and objective radiological evidence of contralateral lesions, rather than routine re‐exploration. This case therefore highlights the importance of individualized multidisciplinary decision‐making and fertility‐preserving surgical planning in adolescent patients.

Plasmapheresis also played an important supportive role during the prolonged disease course. Although definitive control of the disease was ultimately achieved only after complete tumor removal, plasma exchange likely contributed to reducing circulating autoantibody burden while the underlying antigenic source remained unidentified. Rather than following a predetermined schedule, the duration and frequency of plasmapheresis should be individualized according to the patient′s neurological response and overall treatment trajectory.

Finally, although the patient achieved substantial neurological recovery, formal neuropsychological assessment still demonstrated mild residual cognitive impairment at follow‐up. This finding underscores that apparent clinical recovery may not fully reflect cognitive outcomes and highlights the value of structured long‐term neuropsychological assessment in survivors of anti‐NMDAR encephalitis.

## 4. Conclusion

In conclusion, the main clinical message of this case is that persistent anti‐NMDAR encephalitis after initial tumor removal should prompt renewed search for an occult or contralateral ovarian antigenic source, rather than assuming delayed immunologic recovery alone. The case also highlights an important management dilemma in adolescents: Adequate lesion control must be balanced against preservation of future reproductive potential through careful multidisciplinary planning.

**Nomenclature**
Anti‐NMDAR: anti–N‐methyl‐D‐aspartate receptor
CSF: cerebrospinal fluid
CT: computed tomography
IFA: indirect immunofluorescence antibody
IgG: immunoglobulin G.

## Author Contributions

T‐M.T.H. and N‐T.T.N.: writing—original draft. T‐M.T.H., A‐T.T.N, K‐T.N.H., T‐V.P., and N‐K.T.D.: investigation and formal analysis. D‐T.T.H. and Q‐H.L.: writing—review and editing. All authors edited and approved the contents of the submitted manuscript.

## Funding

No funding was received for this manuscript.

## Ethics Statement

According to institutional regulations, this case report was exempt from Institutional Review Board approval, and formal ethical review was therefore waived. Written informed consent for publication of this case report and any accompanying images was obtained from the patient′s mother. A copy of the signed consent form is available for review by the editor‐in‐chief of this journal.

## Conflicts of Interest

The authors declare no conflicts of interest.

## Data Availability

The data that support the findings of this study are available on request from the corresponding author. The data are not publicly available due to privacy or ethical restrictions.
